# Paradoxically Low Levels of Total and HMW Adiponectin in Relation to Metabolic Parameters in a Tongan Population

**DOI:** 10.1155/2013/873507

**Published:** 2013-07-09

**Authors:** Philip Peake, Stephen Colagiuri, Lesley V. Campbell, Yvonne Shen

**Affiliations:** ^1^Renal Laboratory, Department of Medicine, University of New South Wales, Randwick, NSW 2031, Australia; ^2^Institute of Obesity, Nutrition and Exercise, Sydney University, Darlington, NSW 2006, Australia; ^3^Garvan Institute of Medical Research, Sydney University, Darlinghurst, NSW 2010, Australia; ^4^Royal North Shore Hospital, Sydney University, St Leonards, NSW 2065, Australia

## Abstract

*Aim*. Adiponectin has demonstrated anti-inflammatory and insulin sensitising properties, and low circulating levels may be an important risk factor for diabetes. We examined levels of adiponectin and its insulin-sensitising HMW isoform and their relationship with metabolic parameters in Tongans, a population prone to type II diabetes. *Methods*. Adiponectin and its HMW isoform were quantitated by Elisa in specimens from a randomly recruited, multistage cluster population survey of Tongans and from a group of Caucasians. Anthropometric, clinical, and biochemical data were collected on each subject. *Results*. Both male and female Tongans had lower levels of total and HMW adiponectin than their Caucasian counterparts. Levels of total and HMW adiponectin were higher in females than males in each group. Adiponectin levels were inversely related to BMI, weight, and HOMA in Tongan males and females, as well as to dyslipidemia in both sexes. *Conclusion*. Tongans had lower levels of both total and HMW adiponectin than Caucasians population, even after matching Tongans to their Caucasian counterparts based on BMI, age, and sex. These findings may reflect differences in body composition between the populations not adequately assessed by BMI, lifestyle factors, or a genetic variant likely in a genetically homogenous population.

## 1. Introduction

Insulin resistance is a major risk factor for type II diabetes and commonly associated with obesity and a high fat diet. The adipose-specific glycoprotein adiponectin, one of several adipokines produced by adipose tissue, promotes insulin sensitivity, protects against atherosclerosis, and has anti-inflammatory properties [1]. Adiponectin's HMW isomer is the most important correlate of insulin sensitivity [2], and mutant forms of adiponectin unable to form HMW species are associated with diabetes in man [3]. Adiponectin has also been shown to activate different pathways in vitro according to its oligomeric state [4], while the two receptors for adiponectin, adiponectin receptor 1 (AdipoR1) and AdipoR2, display differing functionalities [5]. In mice, simultaneous disruption of both receptors abolished adiponectin binding and resulted in increased levels of tissue triglyceride, inflammation, insulin resistance, and marked glucose intolerance [6] demonstrating the important role for adiponectin in the regulation of glucose and lipid metabolism. Levels of adiponectin are decreased in the obese, those with coronary artery disease, and in type II diabetes [1].

The Tongan population is an ideal group to examine the pathophysiology of diabetes, because the population has a very high prevalence of obesity. Approximately 84% of Tongan men and 93% of Tongan women are classified as either overweight or obese, with both sexes having high levels of type II diabetes [7]. However, while Tongan men have higher weight than their Australian Caucasian counterparts, they do not have significantly more total or abdominal fat. This is largely due to a higher lean mass in Tongan men, with a similar observation made in Tongan women [8]. Such differences in body fat could affect the relationship between adiponectin and obesity/insulin resistance in the Tongan population. The present study was designed to test this idea by examining the interrelationship between adiponectin, body mass index, and ethnicity. We therefore hypothesized that Tongans have lower adiponectin levels than Caucasians and analysed levels of total and HMW adiponectin in a randomly chosen group from the Tongan population in comparison with a group of Caucasian control subjects.

## 2. Materials and Methods

### 2.1. Study Participants

The study was designed as a comparative cross-sectional investigation of two ethnicities: Tongans and Australians of Caucasian background. The Tongan group was randomly recruited from a multistage cluster population in a published survey [7, 9]. The survey and sample collection was approved by Ministry of Health in Tonga, and all subjects gave informed consent in a standardised manner. The Caucasian control group consisted of healthy volunteers without clinical or laboratory evidence of renal disease or diabetes [10]. The study was approved by the Prince of Wales Human Research Ethics Committee, and written informed consent was obtained from each subject. The study conforms to the Code of Ethics of the World Medical Association (Declaration of Helsinki).

### 2.2. Measurements

The height (to the nearest millimeter) and weight (in kilograms) of participants were measured without shoes and in light clothing, and BMI was calculated as weight in kg divided by height in metres squared. Waist (measured at the midpoint between the lower border of the ribs and the iliac crest) and hip (at the widest point over the buttocks) circumferences were measured, and waist-to-hip ratio (WHR) was calculated.

Venous blood sample was taken after an overnight fast and stored at −20°C until analyses. Measurements were made of plasma cholesterol, triglycerides, and high density lipoprotein (HDL), as well as glucose, insulin, and plasma total and HMW adiponectin. IR was estimated using the Homoeostasis Model Assessment index (HOMA-R), that is, (fasting glucose (mmol/L) × fasting insulin (mU/L)/22.5). HbA1c was measured using the DCA 2000 analyzer (Bayer, Elkhart, IN) (normal range 4–6%). All biochemical assays were performed in the NATA-accredited routine diagnostic laboratory of the Prince of Wales Hospital, Sydney.

Adiponectin isoforms are stable in vivo [11] and on long-term storage at −20°C or below [9, 12]. Levels of total and HMW adiponectin levels were measured by commercial Elisa (R&D) (Alpco). Assays on Tongan and control populations were performed on the same kit lot numbers. The inter assay variability CV% was <7% for total adiponectin and <6% for the HMW adiponectin assay.

### 2.3. Statistical Analyses

Data were expressed as mean ± SD. In general, the data obtained were not normally distributed, and an unpaired, two-tailed Mann-Whitney *U* test was used to assess the significance of differences between the populations. To assess the relationship between levels of total and HMW adiponectin and subject characteristics and biochemical parameters, a Spearman correlation coefficient was determined. Levels of total and HMW adiponectin were log transformed to evaluate their interrelationship, and ANCOVA was performed with log adiponectin as the dependent variable to examine the relationship between adiponectin, BMI, gender, and ethnicity.

*P* < 0.05 was considered statistically significant.

## 3. Results

The study included 255 Tongans and 43 Caucasians, whose demographic and lipid parameters are shown in Table 1. In men, there were no significant differences in age and cholesterol levels. In women, Tongans had greater BMI and WHR than their Caucasian counterparts. In contrast to Caucasians, female Tongans had a higher HOMA than male Tongans.

**Figure 1:**
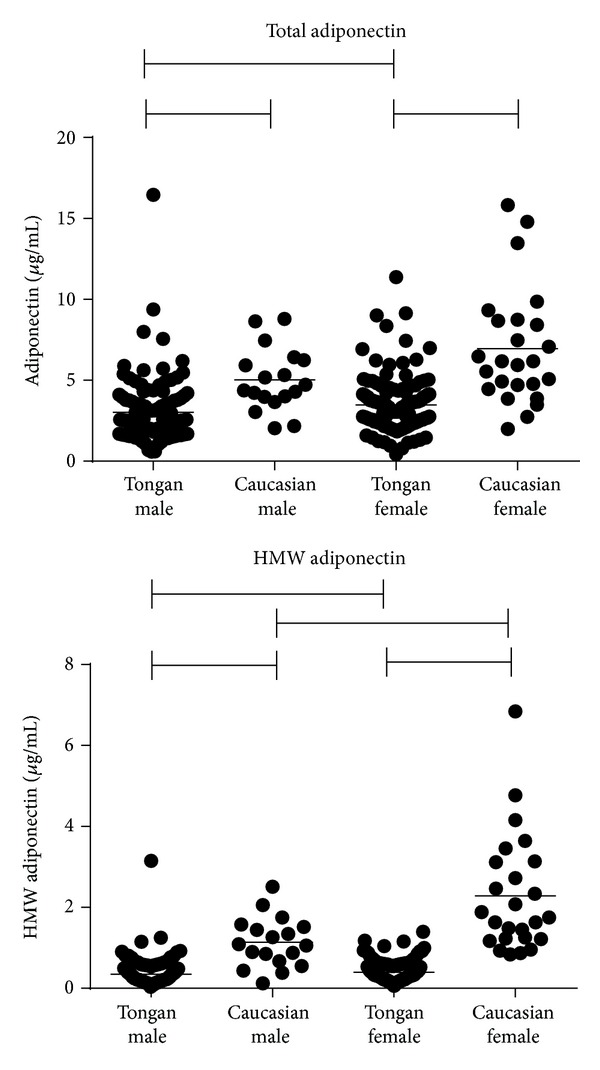
Plasma total and HMW adiponectin levels in Tongan and Caucasian populations. The mean is shown. Lines show significant differences between populations.

Levels of adiponectin were not normally distributed in the Tongan population (Figure 1), but nonparametric analysis showed both male and female Tongans had much lower levels of both total and HMW adiponectin and a lower ratio of HMW : total adiponectin than the Caucasian controls. When the log of HMW adiponectin was compared with log total adiponectin, there were significant linear correlations between these variables in Caucasian males and females (*r* ^2^ = 0.55, *P* < 0.001; *r* ^2^ = 0.38, *P* < 0.001, resp.) and lesser *r* ^2^ values in Tongan males and females (*r* ^2^ = 0.04, *P* < 0.05; *r* ^2^ = 0.12, *P* < 0.001, resp.).

Total adiponectin levels were negatively correlated with weight, BMI, and HOMA in both male and female Tongans, and negatively with WHR in Tongan females (Table 2). In Tongan females, HMW adiponectin was also negatively correlated with HOMA. Total and HMW adiponectin were correlated positively with HDL in Tongan males and females, while in Tongan males both total and HMW adiponectin were negatively correlated with triglyceride levels. 

A linear regression model of the interaction between log adiponectin and the predictor variables of log BMI, and sex (Table 3) confirmed that BMI and sex explained significantly more of the variance in both total and HMW adiponectin in Caucasians than in Tongans.

 Using the strict criterion of HbA1c >6%, we found in Tongans that 10.7% (*n* = 27/253) were diabetic, and these had significant (*P* < 0.05) lower adiponectin after adjusting for BMI and sex in the analysis of covariance model. On average, the adjusted adiponectin in diabetic individuals was 15% (95% CI 0 to 39%) lower than nondiabetic individuals. Total adiponectin was negatively correlated with HbA1c data in male Tongans, and in female Tongans the correlation just failed to reach significance.

To attempt strict adiposity and age matching of groups, a number of Tongans were matched to the Caucasian control group based upon their sex, age, and BMI (Table 1). The males among these age and BMI matched Tongans had a lower WHR and HOMA than their Caucasian counterparts but with significantly lower levels of both total and HMW adiponectin. The female Tongans age and BMI matched to the Caucasian controls also had lower HOMA values, as well as lower total and HMW adiponectin levels.

## 4. Discussion

These results demonstrate paradoxically low levels of both total and HMW adiponectin in both male and female Tongans in comparison with a control population of Caucasians. The latter were matched for age and were overweight (BMI > 25), but it was not possible to match these Caucasians to the obese Tongan population, many of whom had a BMI >30 [7]. Despite this, the WHR in Tongan males was less than in their Caucasian counterparts, while that in Tongan women did not differ significantly from the leaner Caucasian women. Also, there were no significant differences in HOMA and cholesterol between males and females between Tongans and controls. These population data reflect previous studies [8, 13] which noted that Tongan males (reported BMI, 32.8 ± 4.6) were only “fatter” than Australian males by BMI estimation (BMI, 27.1 ± 3.7), while any differences in total body percent fat (28.9 ± 8.3 versus 25.9 ± 8.1), abdominal fat (1.84 ± 0.69 versus 1.55 ± 0.60 kg,) and abdominal percent fat (30.3 ± 8.6 versus 28.5 ± 8.3) were nonsignificant. These studies also showed that Tongan females (reported BMI, 34.3 ± 5.5) were also “fatter” by BMI than their Australian counterparts (BMI, 26.2 ± 6.3) although the extent of the difference in total body percent fat (41.9 ± 5.2 versus 38.7 ± 8.9, *P* = 0.05) and abdominal percent fat (39.3 ± 4.8 versus 33.6 ± 8.9, *P* = 0.001) did not reflect the larger differences in BMI. Notably, Caucasians had a significantly lower lean body and higher abdominal fat masses than Tongans with the same BMI [8, 13] highlighting underlying body composition differences not detectable by BMI comparisons.

There were much lower levels of total and HMW adiponectin in Tongans than in overweight Caucasians, as well as a significantly lower ratio of HMW/total adiponectin. This ratio is an important correlate of insulin sensitivity [2] In both Tongan males and females, total adiponectin was found to correlate negatively with weight, BMI, and HOMA. In other populations, HMW adiponectin has been found to correlate negatively with BMI [14], but we did not detect such a relationship in Tongans. Importantly, in a subset of Tongans matched by sex, age, and BMI to the Caucasian controls, the Tongans had still lower total and HMW adiponectin levels. In addition, the differences in adiponectin levels between Caucasians and Tongans remained after adjustment for BMI in a multivariate analysis.

Adiponectin was also found to be lower in Pima Indians, another indigenous population with a high prevalence of obesity and diabetes [15]. However, their HMW/Total adiponectin ratio did not correlate with HOMA or BMI. Such findings reflect divergent reports in the literature on the utility of HMW and the HMW/total ratio in predicting insulin sensitivity [2, 16]. A lack of correlation between HMW adiponectin and traits of the metabolic syndrome is also seen in African Americans [17]. In general, higher adiponectin levels are associated with better glycaemic control and a better lipid profile [18], results which mirror our findings in Tongan men. 

One notable feature of the Tongan population is its genetic homogeneity [7, 19]. PPARGC1A is a candidate “thrifty gene” promoting the storage of fat and energy in Pacific populations, and the Gly482Ser genotype is associated with BMI in Tongans but not in the Maori [20]. Polymorphisms at the adiponectin locus are predictors of total and HMW adiponectin levels [21–23]. A similar effect may be seen in the Maori population which has low adiponectin levels [24]. 

Diet may contribute to the low levels of adiponectin in Tongans, who show an increasing preference for energy dense foods [7, 25, 26]. A diet rich in monounsaturated fat was found to be associated with higher levels of total and HMW adiponectin in comparison to a carbohydrate- or protein-rich diet [27]. We have shown that a high fat meal caused 23% postprandial downregulation of AdipoR1 expression in humans [28], and the adiponectin receptor is reported to stimulate ceramidase [29] which provides a potential pathway of influencing insulin resistance. In adiponectin-knockout mice, glucose tolerance was significantly decreased on a high-fat diet [30], while adiponectin-receptor knockout mice show marked glucose intolerance [6].

In conclusion, the Tongan population has been reported to have a novel body composition with high lean mass in comparison to the control Caucasian group [8]. This fact may influence the low total and HMW adiponectin levels we noted. As well, genetic homogeneity with the presence of “thrifty” gene(s) and disproportionately low levels of adiponectin and HMW adiponectin may contribute to the high incidence of type II diabetes.

## Figures and Tables

**Table 1:** Clinical and biochemical characteristics of the three study groups.

	Caucasian	Tongan	Matched Tongan
Men			
Number of subjects	18	123	18
Age (yr)	46.7 ± 14.0	52.5 ± 15.9	47.7 ± 14.4
BMI (kg/m^2^)	27.5 ± 3.2	31.0 ± 8.9^b^	27.2 ± 2.8
Waist-to-hip ratio	0.94 ± 0.09	0.90 ± 0.07^a^	0.87 ± 0.07^b^
HOMA	2.6 ± 1.9	2.3 ± 3.0^a^	1.2 ± 0.7^b^
Total cholesterol (mmol/L)	5.1 ± 1.1	5.1 ± 1.0	5.1 ± 1.2
HDL cholesterol (mmol/L)	1.2 ± 0.3	1.1 ± 0.3	1.2 ± 0.3
Triglycerides (mmol/L)	1.4 ± 0.8	1.7 ± 1.3	1.5 ± 1.1
HbA1c (%)	—	5.5 ± 1.1	5.3 ± 0.5
Adiponectin ug/mL	5.0 ± 1.9	3.0 ± 1.9^b^	3.0 ± 2.2^b^
HMW adiponectin ug/mL	1.13 ± 0.62	0.35 ± 0.34^b^	0.59 ± 0.71^b^
Ratio HMW/total adiponectin	0.22 ± 0.06	0.15 ± 0.22^b^	0.36 ± 0.48

Women			
Number of subjects	25	132	25
Age (yr)	46.7 ± 12.1	51.3 ± 15.3	46.3 ± 16.0
BMI (kg/m^2^)	26.2 ± 4.6	34.8 ± 10.7^a,c^	26.7 ± 4.0
Waist-to-hip ratio	0.81 ± 0.06^a^	0.83 ± 0.06^a^	0.81 ± 0.06^a^
HOMA	2.3 ± 1.4	3.1 ± 3.3^a^	1.6 ± 0.8^c^
Total cholesterol (mmol/L)	5.5 ± 1.3	5.0 ± 1.0	4.9 ± 1.1
HDL cholesterol (mmol/L)	1.5 ± 0.4^a^	1.1 ± 0.2^c^	1.2 ± 0.3^c^
Triglycerides (mmol/L)	1.1 ± 0.7	1.2 ± 0.8^a^	1.0 ± 0.5
HbA1c (%)	—	5.4 ± 1.0	5.2 ± 0.4
Adiponectin ug/mL	7.0 ± 3.6	3.5 ± 1.7^a,c^	4.1 ± 2.2^a,c^
HMW adiponectin ug/mL	2.28 ± 1.45^a^	0.40 ± 0.25^a,c^	0.50 ± 0.32^c^
Ratio HMW/total adiponectin	0.36 ± 0.23^a^	0.13 ± 0.11^c^	0.14 ± 0.11^c^

Study groups were healthy Caucasians, a randomly chosen population of Tongans, and a subset of the Tongan group chosen to match the Caucasians in age and BMI.

M versus F for Caucasians and for Tongans.

^a^ *P* < 0.05.

Caucasian M versus Tongan M.

^b^ *P* < 0.05.

Caucasian F versus Tongan F.

^c^ *P* < 0.05.

**Table 2:** Significant Spearman correlation coefficients between total and HMW adiponectin and group characteristics of Tongans.

	Tongan female	*P* value	Tongan male	*P* value
Total adiponectin				
BMI	−0.24	<0.01	−0.19	<0.05
Weight	−0.19	<0.05	−0.20	<0.05
WHR	−0.37	<0.001		
HOMA	−0.23	<0.01	−0.21	<0.05
CHOL	−0.18	<0.05		
HDL	0.18	<0.05	0.18	<0.05
TRIG			−0.37	<0.001
HbA1c			−0.18	<0.05

HMW adiponectin				
HOMA	−0.32	<0.001		
HDL	0.26	<0.01	0.20	<0.05
TRIG			−0.26	<0.01

**Table:** (a) Total adiponectin

Tongans	Estimate	SE	*P* value
Intercept	2.0652	0.75687	<0.01
Log BMI	−0.32216	0.22163	0.15
Sex	0.31754	1.0268	0.75
Log BMI: sex	−0.032921	0.29636	0.91

*R* ^2^ = 0.047, *F*-test *P* < 0.01.

**Table:** (b)

Caucasians	Estimate	SE	*P* value
Intercept	3.2174	2.9265	0.28
Log BMI	−0.50709	0.88467	0.60
Sex	3.1964	3.3648	0.35
Log BMI: sex	−0.9068	1.0213	0.38

*R* ^2^ = 0.24, *F*-test *P* < 0.01.

**Table:** (c) HMW adiponectin

Tongans	Estimate	SE	*P* value
Intercept	0.35596	1.0317	0.73038
Log BMI	−0.49238	0.30212	0.10441
Sex	−0.47322	1.3997	0.73559
Log BMI: sex	0.21042	0.40399	0.60292

*R* ^2^ = 0.038, *F*-test *P* = 0.02.

**Table:** (d)

Caucasians	Estimate	SE	*P* value
Intercept	0.18134	4.2765	0.96639
Log BMI	−0.0734	1.2928	0.95501
Sex	3.5981	4.917	0.46868
Log BMI: sex	−0.88647	1.4924	0.55596

*R* ^2^ is 0.28, *P* < 0.01.
